# Complete genome sequence of *Vibrio vulnificus* FORC_017 isolated from a patient with a hemorrhagic rash after consuming raw dotted gizzard shad

**DOI:** 10.1186/s13099-016-0104-6

**Published:** 2016-06-20

**Authors:** Han Young Chung, You-Tae Kim, Suyeon Kim, Eun Jung Na, Hye-Jin Ku, Keun Hwa Lee, Sang Taek Heo, Sangryeol Ryu, Heebal Kim, Sang Ho Choi, Ju-Hoon Lee

**Affiliations:** Department of Agricultural Biotechnology, Center for Food Safety and Toxicology, Seoul National University, Seoul, Republic of Korea; Department of Food Science and Biotechnology, Kyung Hee University, Yongin, Republic of Korea; Department of Internal Medicine, Jeju National University School of Medicine, Jeju, Republic of Korea; Department of Animal Science and Biotechnology, Seoul National University, Seoul, Republic of Korea

**Keywords:** *Vibrio vulnificus*, Genome sequencing, Hybrid assembly, Pathogenesis, Virulence factor, Hemolysin, Secretion system

## Abstract

**Background:**

*Vibrio vulnificus*, a resident in the human gut, is frequently found in seafood, causing food-borne illnesses including gastroenteritis and severe septicemia. While *V. vulnificus* has been known to be one of the major food-borne pathogens, pathogenicity and virulence factors are not fully understood yet. To extend our understanding of the pathogenesis of *V. vulnificus* at the genomic level, the genome of *V. vulnificus* FORC_017 isolated from a female patient experiencing a hemorrhagic rash was completely sequenced and analyzed.

**Results:**

Three discontinuous contigs were generated from a hybrid assembly using Illumina MiSeq and PacBio platforms, revealing that the genome of the FORC_017 consists of two circular chromosomes and a plasmid. Chromosome I consists of 3,253,417-bp (GC content 46.49 %) containing 2943 predicted open reading frames (ORFs) and chromosome II of 1,905,745-bp (GC content 46.90 %) containing 1638 ORFs. The plasmid pFORC17 consists of 70,069-bp (GC content 43.77 %) containing 84 ORFs. The average nucleotide identity (ANI) value of the FORC_017 and CMCP6 strains was 98.53, suggesting that they are closely related.

**Conclusions:**

Pathogenesis-associated genes including *vvhA*, *rtx* gene cluster, and various hemolysin genes were present in FORC_017. In addition, three complete secretion systems (Type I, II and VI) as well as iron uptake-related genes for virulence of the FORC_017 were detected, suggesting that this strain is pathogenic. Further comparative genome analysis revealed that FORC_017 and CMCP6 share major toxin genes including *vvhA* and *rtx* for pathogenesis activities. The genome information of the FORC_017 provides novel insights into pathogenicity and virulence factors of *V. vulnificus*.

**Electronic supplementary material:**

The online version of this article (doi:10.1186/s13099-016-0104-6) contains supplementary material, which is available to authorized users.

## Background

*Vibrio vulnificus* infections in humans via either the consumption of contaminated seafood or contact of a wound with seawater generally cause mild gastroenteritis with diarrhea, vomiting, and abdominal pain [1]. In addition, this pathogen in the human gut can cause life-threatening systemic septicemia with a mortality rate of 61 % when it crosses the intestinal mucosal barrier to enter the bloodstream, suggesting that *V. vulnificus* is one of many pathogenic bacteria in the human gut microbiota [2, 3]. *Vibrio vulnificus* has been frequently detected in sea water as well as various kinds of seafood [4]. *Vibrio vulnificus* was detected in 66 of 68 oyster samples (97 %) and 27 of 30 clam samples (90 %) from Long Island Sound, California [5]. The consumption of *V. vulnificus*-contaminated seafood causes clinical food-borne outbreaks. In South Korea (2000–2011), 34 individuals who consumed *V. vulnificus*-contaminated seafood were hospitalized and 16 died, which suggests that this pathogen is widespread and highly virulent and causes food-borne outbreaks [6]. Therefore, virulence traits of this pathogen should be understood at the genomic level. Although outbreaks of this pathogen have been reported for many years, only five strains of *V. vulnificus* have had their whole genomes sequenced and analyzed to date (NCBI; http://www.ncbi.nlm.nih.gov). Genome information has revealed the presence of various virulence factors in the genomes, such as an extracellular hemolysin/cytolysin, iron uptake receptors, capsular polysaccharide related to antiphagocytosis, and secretion systems [7]. To extend our understanding of the pathogen’s further pathogenicity and virulence factors at the genomic level, more genomes of *V. vulnificus* need to be sequenced and analyzed.

To elucidate the pathogenesis of *V. vulnificus* in South Korea, a clinical strain, *V. vulnificus* FORC_017, was isolated from a 63-year-old woman with the symptom of a hemorrhagic rash after consuming a raw dotted gizzard shad on Jeju Island. The genome was completely sequenced and analyzed using various bioinformatics tools. In addition, comparative genome analysis of the FORC_017 strain and other clinical isolates was performed. The results of this study would provide genomic insights into the main factors associated with food-borne outbreaks and pathogenesis, and is probably useful for further control of this highly virulent pathogen in seafood.

## Methods

### Growth condition and genomic DNA extraction

The FORC_017 strain was cultivated aerobically at 30 °C with modified Luria-Bertani medium (LB) supplemented with 1 % (w/v) NaCl for 12 h. The genomic DNA was extracted using DNeasy Blood & Tissue Kit (Qiagen, Valencia, CA, USA), according to the manufacturers protocol.

### Genome sequencing and annotation

The whole genome sequencing and assembly were conducted at ChunLab, Inc. (Seoul, South Korea). Sequences of *V. vulnificus* FORC_017 were obtained using two different platforms of PacBio RS II platform (Pacific Biosciences, Menlo Park, CA, USA) and Illumina MiSeq platform (Illumina, San Diego, CA, USA). The sequencing results were summarized in Additional file 1: Table S1. The qualified sequence reads were assembled with CLC Genomic Workbench 7.5.1 (CLC Bio, Aarhus, Denmark). The prediction of open reading frames (ORFs) and their annotations were performed using the GeneMarkS program and the Rapid Annotations using Subsystems Technology (RAST) server [8, 9]. The prediction of ribosome binding sites (RBS) was conducted using the RBSfinder (J. Craig Venter Institute, Rockville, MD, USA). Subsequent predictions of the functions of ORFs and their conserved protein domains were carried out using InterProScan 5 and COG-based WebMGA programs [10, 11]. The putative virulence factors of the *V. vulnificus* FORC_017 strain were predicted and characterized using the basic local alignment search tool (BLAST) in the Virulence Factor Database (VFDB; http://www.mgc.ac.cn/VFs/main.htm). The circular genome maps of the FORC_017 strain were drawn using the GenVision program (DNASTAR, Madision, WI, USA).

### Comparative genome analysis

The strain was identified as *V. vulnificus* using 16S rRNA sequencing and its identity was confirmed with comparative phylogenetic tree analysis using MEGA6 [12] with various 16S rRNA sequences of the genus *Vibrio*. Phylogenetic trees were constructed from the aligned sequences using the neighbor-joining method with 1000 bootstrap replicates via MEGA6 software. *Shewanella baltica* OS678 was used as the out-group. ANI phylogenetic tree analysis of complete genome sequences of *V. vulnificus* (93U204, YJ016, CMCP6, FORC_016 and MO6-24/O) was conducted using the JSpecies program [13] to show the evolutionary relationship among *V. vulnificus* strains. The ANI values were calculated using JSpecies by comparing whole genome sequences between *V. vulnificus* strains, which were fragmented into 1020-bp, based on BLAST algorism. The tree was constructed using the R program. The comparative genome analysis was conducted with the Artemis Comparison Tool (ACT) [14].

### Quality assurance

A single colony of the FORC_017 strain was isolated and its morphology was observed using a transmission electron microscopy showing a traditional shape of *V. vulnificus*, including a short rod with a single flagellum (Additional file 1: Figure S1). The genomic DNA of the FORC_017 strain was extracted from the isolate and confirmed to *V. vulnificus* by 16S rRNA gene sequencing. The 16S rRNA genes were amplified using PCRwith a 16S rRNA universal primer set and the PCR products were sequenced using capillary sequencing technology for taxonomic identification [15]. In addition, when the quality scores of the raw read sequences from NGS sequencers were more than 40 as cutoffs, they were selected and assembled. The assembled genome sequence was also verified with comparative ANI analysis with the published complete genome sequences of the same species.

## Initial findings

### Genome properties

**Fig. 1:**
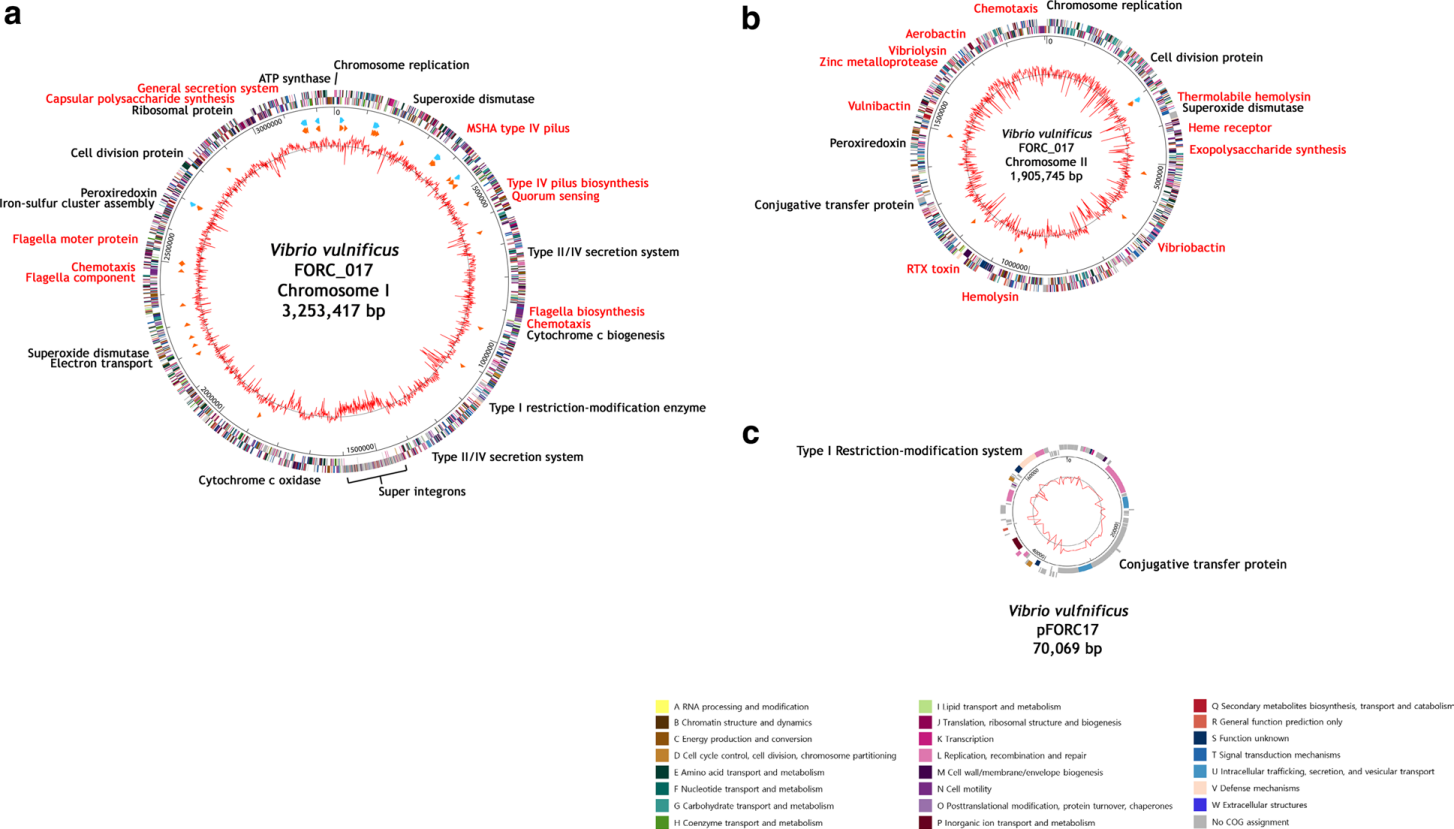
The circular genome maps of the strain FORC_017 using the GenVision program (DNASTAR, Madison, WI, USA). **a** Chromosome I. **b** Chromosome II. **c** Plasmid FORC_017. The *outer circle* indicates the locations of all annotated ORFs and the *inner circle* with the *red peaks* indicates GC content. Between these circles, *sky blue arrows* indicate the rRNA operons and *orange arrows* indicate the tRNAs. All annotated ORFs were colored differently according to the COG assignments. Genes with specialized functions labeled with different colors as follows; virulence-related genes in *red* and other functional genes in *black*

The hybrid platforms of MiSeq and PacBio with a total fold coverage of 438.12 presented that the complete genome of *V. vulnificus* FORC_017 consists of two circular DNA chromosomes and a plasmid. Chromosome I consists of 3,253,417-bp with a GC content of 46.49 % containing 2943 predicted ORFs, 102 tRNA genes, and 31 rRNA genes. Among the ORFs, 2326 ORFs (79.03 %) were predicted to be functional and 617 ORFs (20.96 %) to encode hypothetical proteins. Chromosome II consists of 1,905,745-bp with a GC content of 46.90 % containing 1638 predicted ORFs, 13 tRNA genes, and 3 rRNA genes. Among the ORFs, 1256 ORFs (76.68 %) were predicted to be functional and another 382 ORFs (23.32 %) to encode hypothetical proteins. The plasmid, pFORC17, consists of 70,069-bp with a GC content of 43.77 % containing 84 predicted ORFs. 36 ORFs (42.86 %) were predicted to be functional and another 48 ORFs (57.14 %) to encode hypothetical proteins. Based on the bioinformatics analysis of these chromosomes and a plasmid, circular genome maps were drawn (Fig. 1).

### Pathogenesis and virulence factors

Key virulence factors in *V. vulnificus* are a hemolysin/cytolysin (VvhA) exotoxin and a repeats-in-toxin (Rtx) (Additional file 1: Table S2) [16]. The genome of the FORC_017 strain has a *vvhA* gene (FORC17_3853) and an *rtx* gene cluster (FORC17_3923, 3924, 3926 and 3927), suggesting that FORC_017 is pathogenic. The FORC_017 strain has various hemolysin genes such as FORC17_0475, 1369, 2364, 2546, 2587 and 2903 in chromosome I, and FORC17_3057, 3239 and 4284 in chromosome II. Subsequent cytotoxicity test of the FORC_017 strain supported its pathogenesis activity (Additional file 1: Figure S2).

Various secretion systems including Types I, II, IV and VI (T1SS, T2SS, T4SS and T6SS) were observed in the genome sequences of the FORC_017 strain. T1SS secretes gram-negative bacterial RtxA exotoxins in *Vibrio*, which play a role in pore formation in mammalian cells to cause death [17]. T1SS was detected in the specific region of chromosome II (FORC17_4414 to FORC17_4539). The presence of *rtx* gene cluster and T1SS suggests the pathogenesis of the FORC_017 strain toward the human cells. T2SS secretes bacterial cholera-like toxins as well as proteases and lipases. This T2SS was found in the specific region of chromosome I (FORC17_2808 to FORC17_2819). However, the FORC_017 strain does not have this cholera-like toxin in the genome. Therefore, T2SS may be associated with protease/lipase secretions, which is probably related to the damage of the membrane in the human cells for infection of *V. vulnificus* [18]. While T4SS is generally known to be associated with the translocation of bacterial virulence factors into human cells [19], the FORC_017 strain has only one gene encoding VirD2 (FORC17_3580), suggesting that the FORC_017 strain does not have a completely functional T4SS for translocation of virulence factors. T6SS is known to be associated with bacterial toxin protein injection machinery for virulence to human cells. This T6SS was encoded in a gene cluster of chromosome II (FORC17_3873 to FORC17_3890) [20]. Therefore, these secretion systems may contribute to the virulence toward human cells for food-borne illnesses.

Iron uptake from host cells plays a key role in the survival of *V. vulnificus* since iron loss caused by *V. vulnificus* negatively affects the heme protein integrity, resulting in host cell death [22, 23]. The FORC_017 genome included heme receptors (FORC17_3295 in chromosome II), iron ABC transporters (FORC17_3542 to FORC17_3544 in chromosome II), and vibriobactin receptors (FORC17_4247 to FORC17_4256 in chromosome II). These iron uptake-related genes support the survival of the FORC_017 strain in the host cells.

### Comparative genome analysis of the closely related FORC_017 and CMCP6 strains

**Fig. 2:**
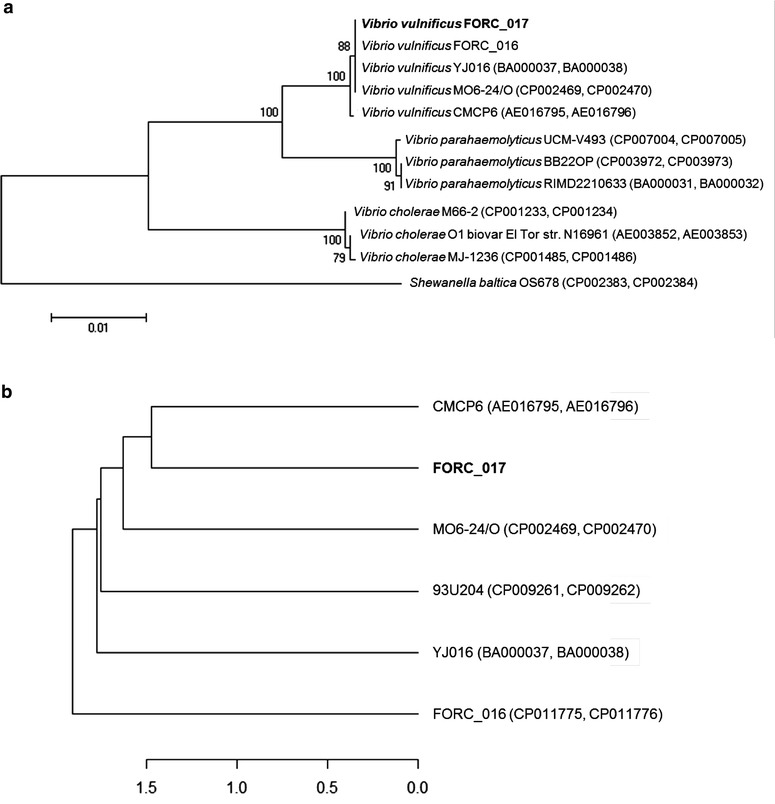
**a** The phylogenetic tree of the FORC_017 strain with closely related *Vibrio* species aligned by using ClustalW. *Shewanella baltica* OS678 was used as the out-group. **b** The ANI genome tree of various *V. vulnificus* strains were constructed using the ANI values. The complete genome sequences of *V. vulnificus* (93U204, YJ016, CMCP6, FORC_016 and MO6-24/O) revealed that the FORC_017 strain has the closest evolutionary relationship (ANI value 98.53) with a clinical isolate, *V. vulnificus* CMCP6, from a patient in South Korea

Comparative phylogenetic tree analysis of the FORC_017 strain and other *Vibrio* strains using 16S rRNA sequences showed that the FORC_017 strain belongs to *V. vulnificus* (Fig. 2a). Further ANI phylogenetic tree analysis of complete genome sequences of *V. vulnificus* (93U204, YJ016, CMCP6, FORC_016 and MO6-24/O) revealed that the FORC_017 strain has the closest evolutionary relationship (ANI value 98.53) with a clinical isolate, *V. vulnificus* CMCP6, from a patient in South Korea (Fig. 2b) [21]. Interestingly, comparative genome analysis of two closely related strains, FORC_017 and CMCP6, showed that the FORC_017 strain has additional virulence factors including thermostable hemolysin delta-VPH (FORC17_3057) ansd partial T4SS (FORC17_3580). Although this thermostable hemolysin may be involved in the pathogenesis toward humans, the presence of a single gene related to T4SS in the FORC_017 strain is not fully understood yet. Probably, this single gene encoding VirD2 may be a remnant of a complete T4SS gene cluster after its deletion event from chromosome II of the FORC_017 strain. The presence of mobile element proteins near VirD2 (FORC17_3591 and 3592) supports this hypothesis.

## Future directions

The genome information of the FORC_017 strain provides genomic insights into its pathogenicity and various virulence factors as well as their virulence mechanisms in *V. vulnificus*, which is probably involved in the seafood-borne outbreaks. In addition, the FORC_017 strain could provide further genomic information for development of rapid detection and control methods against *V. vulnificus* in various kinds of seafood. Therefore, genome information can be applied for rapid detection, epidemiological survey and prevention of food poisoning caused by this strain.

## Additional file

10.1186/s13099-016-0104-6 Summary of *V. vulnificus* FORC_017 genome. **Figure S1.** Transmission electron micrograph of *Vibrio vulnificus* FORC_017. The cells were negatively stained with uranyl acetate (UA) for one minute and were observed using TEM JEM-2100 (JEOL, Tokyo, Japan) at 200 kV. **Table S2.** Virulence factor of *V. vulnificus* FORC_017 using the Virulence Factor Database. **Figure S2.** Cytotoxicity analyses were conducted with *V. vulnificus* FORC_017 and *V. vulnificus* MO6-24/O, which was the positive control. INT-407 cells were infected with FORC_017 at multiplicities of infection (MOIs) for 2 h. Cytotoxicity was determined as the percentage of lactate dehydrogenase (LDH) leakage using the amount of LDH from the cells that were completely lysed by 2 % Triton X-100. Error bars represent the standard error of the mean (SEM).

## Abbreviations

ORF: open reading frame
ANI: average nucleotide identity
TEM: transmission electron microscopy
FORC: Food-borne Pathogen Omics Research Center
VvhA: hemolysin/cytolysin exotoxin
Rtx: a repeats-toxin
LB: Luria-Bertani medium
RAST: Rapid Annotation using Subsystem Technology
RBS: ribosome binding site
ACT: Artemis Comparison Tool
T1SS: type I secretion system
T2SS: type II secretion system
T4SS: type IV secretion system
T6SS: type VI secretion system
