# Supplementary material for: Complete genome sequence of Vibrio vulnificus FORC_017 isolated from a patient with a hemorrhagic rash after consuming raw dotted gizzard shad
Source: Gut Pathog. 2016 Jun 20;8:22. doi: 10.1186/s13099-016-0104-6 (PMC4913425; doi:10.1186/s13099-016-0104-6)
Supplement: Supplementary file 1 — 10.1186/s13099-016-0104-6 Summary of V. vulnificus FORC_017 genome. Figure S1. Transmission electron micrograph of Vibrio vulnificus FORC_017. The cells were negatively stained with uranyl acetate (UA) for one minute and were observed using TEM JEM-2100 (JEOL, Tokyo, Japan) at 200 kV. Table S2. Virulence factor of V. vulnificus FORC_017 using the Virulence Factor Database. Figure S2. Cytotoxicity analyses were conducted with V. vulnificus FORC_017 and V. vulnificus MO6-24/O, which was the positive control. INT-407 cells were infected with FORC_017 at multiplicities of infection (MOIs) for 2 h. Cytotoxicity was determined as the percentage of lactate dehydrogenase (LDH) leakage using the amount of LDH from the cells that were completely lysed by 2 % Triton X-100. Error bars represent the standard error of the mean (SEM). [file 13099_2016_104_MOESM1_ESM.docx]

**Additional Files**

**Complete Genome Sequence of *Vibrio Vulnificus* FORC_017 Isolated from a Patient with a Hemorrhagic Rash after Consuming Raw Dotted Gizzard Shad**

Han Young Chung^­^,^1,†^ You-Tae Kim,^2^ Suyeon Kim,^1^ Eun Jung Na^­,1^ Hye-Jin Ku,^2^ Keun Hwa Lee,^3^ Sang Taek Heo,^3^ Sangryeol Ryu,^1^ Heebal Kim,^4^ Ju-Hoon Lee,^2,*^ Sang Ho Choi^­1,*^

**Author details**

^1^Department of Agricultural Biotechnology, Center for Food Safety and Toxicology, Seoul National University, Seoul, Republic of Korea

^2^Department of Food Science and Biotechnology, Kyung Hee University, Yongin, Republic of Korea

^3^Department of Internal Medicine, Jeju National University School of Medicine, Jeju, Republic of Korea

^4^Department of Animal Science and Biotechnology, Seoul National University, Seoul, Republic of Korea

***Correspondence:**

**Dr. Ju-Hoon Lee:** juhlee@khu.ac.kr

^2^Department of Food Science and Biotechnology, Kyung Hee University, Yongin, Republic of Korea

**Dr. Sang Ho Choi:** choish@snu.ac.kr

^1^Department of Agricultural Biotechnology, Center for Food Safety and Toxicology, Seoul National University, Seoul, Republic of Korea

**Materials and Methods**

**Cytotoxicity test**

Cytotoxicity of FORC_017 was evaluated by measuring the activity of cytoplasmic lactate dehydrogenase (LDH) that is released from the human epithelial INT-407 cells after the plasma membrane was damaged. The INT-407 cells were grown in minimum essential medium containing 1% (v/v) fetal bovine serum (MEMF) (Gibco-BRL, Gaithersburg, MD) in 96-well culture dishes (Nunc, Roskilde, Denmark) as described previously [1]. Each well of the INT-407 2 × 10^4^ cells was infected with FORC_017 and MO6-24/O as a control at various multiplicities of infection (MOIs) for 2h. The LDH activity released into the supernatant was determined using a cytotoxicity detection kit (Roche, Mannheim, Germany).

**References**

1. Kim S, Bang Y-J, Kim D, et al. Distinct characteristics of OxyR2, a new OxyR-type regulator, ensuring expression of Peroxiredoxin 2 detoxifying low levels of hydrogen peroxide in *Vibrio vulnificus*. Mol microbiol. 2014;93:992-1009.

**Table S1. Summary of *V. vulnificus* FORC_017 genome**

| **Property** | **Term** |
| --- | --- |
| Finishing quality | Finished |
| Libraries used | Illumina 300 base pair paired-end library Roche 8 kb paired end library PacBio SMRTbell™ library (> 10kb) for draft assembly |
| Sequencing platforms | Illumina MiSeq_300_PE, PacBio_20K |
| Assemblers | CLCbio CLC Genomics Workbench 7.5.1 Roche gsAssembler 2.6 PacBio SMRT Analysis 2. 3. 0 |
| Gene calling method | RAST ver. 2.0 (Glimmer 3), GeneMarkS |
| Method reads | 7,105,058 (Illumina_Miseq_300_PE)  73,582 (PacBio_20K) |
| Average genome coverage | 438.12x |
| Chromosome length (bp) | 3,253,417 (Chromosome I)  1,905,745 (Chromosome II)  70,069 (Plasmid) |
| Contigs no. | 3 |
| Scaffolds no. | 3 |
| N50 | 3,253,417 |
| Locus Tag | FORC17 |
| Genbank ID | CP012739, CP012740, CP012741 |
| Genbank Date of Release | 2016-08-05 |
| BIOPROJECT | PRJNA291949 |
| Source Material Identifier | FORC_017 |

**Figure S1**


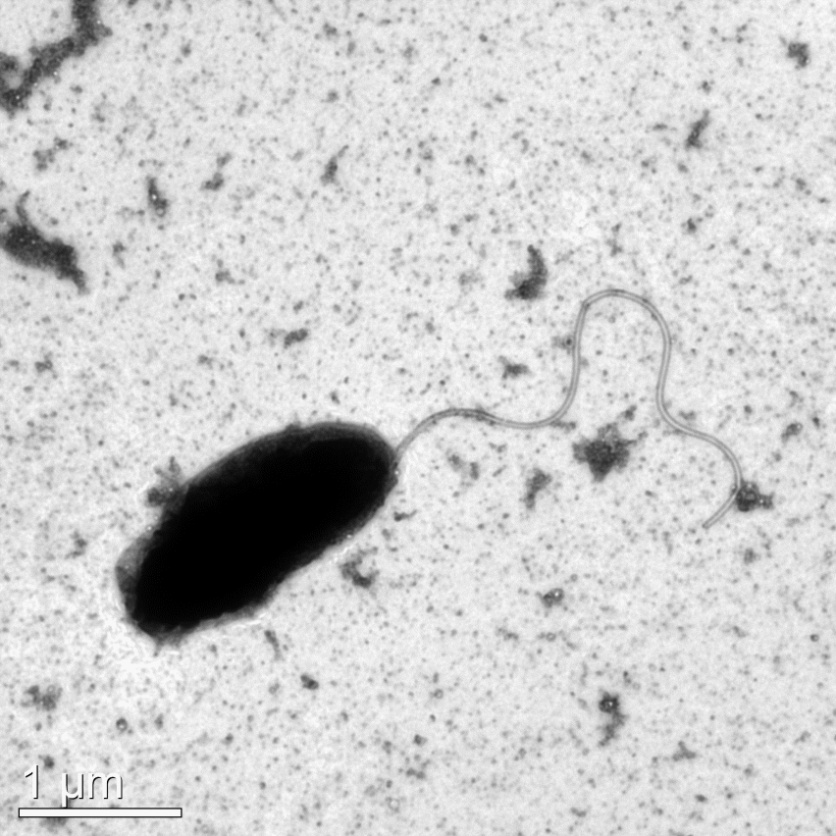


**Additional file 2.**Transmission electron micrograph of *Vibrio vulnificus* FORC_017. The cells were negatively stained with uranyl acetate (UA) for one minute and were observed using TEM JEM-2100 (JEOL, Tokyo, Japan) at 200 kV.

**Table S2. Virulence factor of *V. vulnificus* FORC_017 using the Virulence Factor Database**

| **Virulence factor** | **Annotation** | **Chromosome /Plasmid** | **Location** |
| --- | --- | --- | --- |
| **Adherence** |  |  |  |
| *mshH, mshI, mshJ, mshK, mshL, mshM, mshN, mshE, mshG, mshF, mshB, mshA, mshC, mshD* | Mannose-sensitive hemagglutinin  (MSHA type IV pilus) | Chromosome I | 388661-402365 |
| *pilA, pilB, pilC, pilD* | Type IV pilus | Chromosome I | 564931-569288 |
| **Antiphagocytosis** |  |  |  |
| *wza, hp1, wzb, wzc* | Capsular polysaccharide | Chromosome I | 3012097-3016711 |
| *wcaJ* | UDP-N-acetylgalactosaminyltransferase | Chromosome II | 2999385-2999999 |
| *wbfY, wbfV/wcvB* | Capsular polysaccharide | Chromosome I | 2993980-2997274 |
| *cpsA, cpsB, cpsC, cpsD, cpsF, cpsH, cpsI, cpsJ* | Capsular polysaccharide synthesis enzyme | Chromosome II | 459351-469149 |
| **Chemotaxis and motility** |  |  |  |
| *flaC, flaA, flgL, flgK, flgJ, flgI, flgH,  flgG, flgF, flgE, flgD, flgC, flgB, flgA, flgM, flgN* | Flagella | Chromosome I | 2459365-2473562,  2475837-2477502 |
| *cheR, cheV* | Chemotaxis protein | Chromosome I | 2473994-2475761 |
| *flhB, fliR, fliQ, fliP, fliO, fliN, filM, fliL, fliK, fliJ, fliI, fliH, fliG, fliF, fliE, flrC, flrB, flrA, fliS, flaI, fliD, flaG, flaB, flaD, flaE* | Flagella | Chromosome I | 877422-902661 |
| *cheW, cheB, cheA, cheZ, cheY* | Chemotaxis protein | Chromosome I | 908128-915141 |
| *fliA, flhG, flhF, flhA* | Flagella | Chromosome I | 902822-908095 |
| *motA, motB, motY, motX* | Flagella | Chromosome I | 2555896-2555829, 2231322-2232203  255239-255874 |
| **Exoenzyme** |  |  |  |
| *hap/vvp* | Vibriolysin, extracellular zinc protease | Chromosome II | 1672052-1673881 |
| **Iron uptake** |  |  |  |
| *hutR* | TonB-dependent heme receptor HutR | Chromosome II | 427049-429184 |
| *vctP, vctD, vctG, vctC* | Periplasmic binding protein-dependent ABC transport systems | Chromosome II | 1539661-1540569  694248-696877 |
| *vibB, vibE, vibC, vibA, vibD, vibF* | Vibriobactin biosynthesis | Chromosome II | 1535961-1536848  1530975-1534963  1521107-1521802, 1524930-1529492 |
| *viuB, viuA* | Ferric vulnibactin receptor | Chromosome II | 1535082-1535897  1540656-1542713 |
| **Quorum sensing** |  |  |  |
| *luxS* | Autoinducer-2 | Chromosome I | 556420-556938 |
| **Secretion system** |  |  |  |
| *epsN, epsM, epsL, epsK, epsJ, epsI,  epsH, epsG, epsF, epsE, gspD, epsC* | EPS type II secretion system | Chromosome I | 3095010-3106329 |
| **Toxin** |  |  |  |
| *vvhA* | Hemolysin/cytolysin | Chromosome II | 1071221-1072636 |
| *rtxA, rtxC, rtxB, rtxD* | RTX toxin | Chromosome II | 1152274-1166473  1167364-1170827 |
| *tlh* | Thermolabile hemolysin precursor | Chromosome II | 349482-350735 |

**
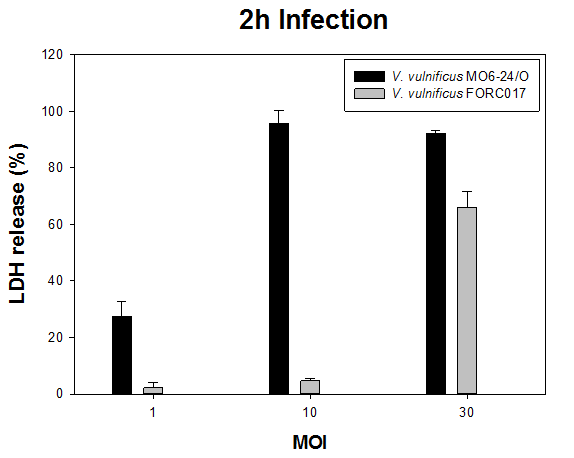
**

**Figure S2.** Cytotoxicity analyses were conducted with *V. vulnificus* FORC_017 and *V. vulnificus* MO6-24/O, which was the positive control. INT-407 cells were infected with FORC_017 at various multiplicities of infection (MOIs) for 2h. Cytotoxicity was determined as the percentage of LDH leakage using the amount of LDH from the cells that were completely lysed by 2% Triton X-100. Error bars represent the standard error of the mean (SEM).
